# Online and in-person stress induction in a quasi-avoidance-based Pavlovian-to-instrumental transfer task

**DOI:** 10.3758/s13420-026-00707-5

**Published:** 2026-02-12

**Authors:** Ruoqi Tang, Jessica M. Price, Charlotte Bonardi

**Affiliations:** 1 School of Psychology, University of Nottingham, University Park, Nottingham, NG7 2RD UK; 2 School of Psychology, University of Nottingham Malaysia, Jalan Broga, 43500 Semenyih, Selangor Darul Ehsan Malaysia

**Keywords:** Pavlovian instrumental transfer, Specific PIT, General PIT, Stress induction, Addiction, Quasi-avoidance

## Abstract

Pavlovian instrumental transfer (PIT) is a phenomenon in which a conditioned stimulus (CS), previously paired with a specific outcome, can influence performance of an instrumental response (R). PIT can be specific (the CS selectively elevates performance of an R paired with the *same* outcome as that CS) or general (CSs elevate performance of an R paired with *any* outcome of the same motivational value). PIT has been proposed as a mechanism underlying maintenance of addictive behaviours, while exposure to stressors is regarded as a risk factor for the development, maintenance and relapse of addiction. Thus, we used a quasi-avoidance-based PIT task to explore whether stress can influence the magnitude of specific or general PIT effects. We used stress induction procedures to elevate stress levels. These were either conducted online, exposing participants to negative pictures and heavy metal music (Experiment 1), or in person, telling participants they had to give a speech which would be filmed (Experiment 2). In neither experiment could we detect any effect of higher stress levels on specific or general PIT. The results do not support the proposal that stress influences the size of PIT effects in human participants.

**Supplementary Information:**

The online version contains supplementary material available at https://doi.org/10.3758/s13420-026-00707-5.

## Introduction

*Pavlovian-instrumental transfer* (PIT; Holmes et al., 2010) is a phenomenon illustrating the ability of Pavlovian cues signalling an outcome to elevate levels of instrumentally conditioned responding to obtain that outcome. PIT effects can be *specific—*a stimulus signalling a specific outcome selectively elevates responding for that same outcome, relative to others of the *same* motivational value—or *general*, where a signal for an outcome elevates responding for *any outcome of the same motivational value*, relative to the effect of a stimulus without consequence. These effects can be differentiated by their respective reliance on motivational and sensory aspects of the outcomes, and evidence suggesting they have different neural substrates (Corbit & Balleine, 2005, 2011; Prévost et al., 2012). PIT is thought to play a critical role in the maintenance of addictive behaviour, and PIT tasks are widely used explore addiction-related phenomena (Cartoni et al., 2016).

Many PIT tasks employ a single-outcome procedure, in which a CS+ and a response R+ are paired with an outcome, and a CS− and another response, R−, are nonreinforced. The PIT test then compares the ability of the CS+ and CS− to elevate performance of R+. However, this test cannot discriminate between general and specific PIT, and so more elegant designs have been devised which can identify general and specific PIT independently (Corbit & Balleine, 2005). For example, Quail et al. (2017; cf. Nadler et al., 2011), used a computer-based appetitive PIT task with a cover story of obtaining snacks from a vending machine. Different coloured lights signalled either different kinds of snack (S1→O1, S2→O2, S3→O3), or the word ‘*empty*’ (S4→nothing). Participants also learned that pressing one of two response keys (R1 and R2) each produced one of the outcomes (R1→O1, R2→O2). Quail et al. reported that in an extinction test participants performed R1 more than R2 in the presence of S1, but R2 more than R1 in the presence of S2 (specific PIT), and also performed *both* R1 and R2 more during S3 than S4 (general PIT). As we will see below, a number of studies have examined the effects of *stress* on appetitive PIT, but relatively few have been equipped to disambiguate the effects of stress on general and specific PIT.

Stress is a physiological and emotional response to an external threat, whether real or perceived (Chrousos, 2009; Selye, 1936), and both acute and chronic stress can have varied and profound effects on learning (Giovanniello et al., 2023). To the extent that learning processes underlie addictive behaviour, it is perhaps not surprising that stress and addictive behaviours often co-occur (Goeders, 2003), with high stress being associated with several types of addiction (Loton et al., 2016; Moge & Romano, 2020; Parylak et al., 2011; Samaha & Hawi, 2017; Younes et al., 2016; see also Kouvonen et al., 2005). For example, PTSD is associated with alcohol abuse, eating disorders and gambling disorders (Coughlin et al, 2011; Grubbs et al., 2019; Kessler et al., 2005), and in some cases this association may be causal: in a longitudinal survey of how stress and various other psychosocial factors vary with substance abuse in urban adolescents, Wills (1986) found that stress appeared to be *predictive* of smoking and alcohol use, and that this pattern could not be accounted for by covariation with potential third causes, such as self-esteem, locus of control, or assertiveness. Moreover, rates of substance abuse are higher among people with PTSD than are rates of PTSD in substance abusers (Donovan et al., 2001).

Given the potential relevance of stress to addiction, it is pertinent to ask whether this effect of stress might be mediated by its effect on PIT. In fact, many studies have examined the effects of stress on *appetitive* PIT, but the results are mixed. Many of these studies employed single-outcome PIT tasks, performance on which could reflect general *or* specific PIT. For instance, Peciña et al. (2006) trained rats that a CS+ signalled sucrose and a CS− nothing, and then that they could obtain sucrose by pressing one of two levers (R1→O, R2→nothing). They then received microinjections of either 250 ng or 500 ng of corticotropin-releasing factor, thought to mediate responses to stress, into the caudal medial accumbens shell, before evaluating responding on the two levers in the presence of the two CSs. The rats in the 500 ng CRF group showed enhanced levels of R1, but not R2, during the CS+, but not during the CS−; an apparent *enhancement* of PIT. However, Pielock et al. (2013) reported no effect in a similarly ambiguous single outcome PIT task in which, before the test, rats experienced various stressors (e.g., restraint, tail shock). Although the stressors affected other behaviours, they did not influence PIT. More recently Derman & Lattal (2022, 2024) administered a series of foot shocks to rats either before Pavlovian and instrumental training, or afterwards, just prior to a single-outcome PIT test. Although the pretraining shocks had no reliable effect on either instrumental or classical conditioning, they *impaired* PIT in adult male rats. However, this effect was absent in the post-training shock group (but see Derman & Lattal, 2024), and in male adolescent rats. In contrast, in both adolescent and adult female rats PIT was *enhanced* (Derman & Lattal, 2024).

In face of such inconsistencies, unambiguous tests of specific and general PIT are especially informative. Morgado et al. (2012) conducted a specific PIT task, exposing male rat subjects unpredictably to one of five kinds of stressors every day for a month. Although the stressors influenced the acquisition of neither Pavlovian nor instrumental conditioning, in the transfer test rats in the *stress* group failed to show specific PIT, in contrast to the control animals. However, Derman and Lattal (2022) found that the same pretraining shock treatment that impaired single-outcome PIT had no effect on specific PIT in either men or women.

The effect of stress on appetitive PIT is no clearer in humans. Pool et al. (2015) trained participants on a single outcome task in which participants could obtain chocolate odour by squeezing a handgrip; three different visual cues were paired with chocolate odour, odourless air, or nothing. Before the PIT test participants in the *stress* group were encouraged to put their nondominant hands in cold water for as long as they could (the socially evaluated cold pressor test [SECPT]; Schwabe et al., 2008). In the transfer test stressed participants showed selective elevation of responding during the chocolate-paired stimulus, relative to a *stressfree* group, suggesting an *enhancement* of PIT. However, results with less ambiguous tests of general and specific PIT have failed to reveal such enhancements. Quail et al. (2017), using the Depression Anxiety Stress Scale (DASS; Lovibond, & Lovibond, 1995), found in their vending machine task that participants who scored higher on the combined stress and anxiety subscales showed a *smaller* general PIT effect, while specific PIT was unaffected. However, this reduction in general PIT was driven by higher response rates during S4, paired with an ‘empty’ machine, rather than lower responding to S3. This, in combination with the small sample size of 24 casts some doubt on whether this was a genuine effect on general PIT. Vogel et al. (2018) also found no relationship between scores on the Perceived Stress Questionnaire (PSQ20; Levenstein et al., 1993) and performance on a specific PIT task, in which participants pressed ‘G’ and ‘S’ keys to obtain gaming and shopping points, while Steins-Loeber et al. (2020) found no effect of the SECPT manipulation on a specific PIT task using chocolate and cigarette outcomes (see also Pritchard et al., 2018, who stressed participants with aversive images from the International Affective Picture System (IAPS; Lang et al., 1997).

Overall, then, the picture for appetitive PIT suggests that in single-outcome tasks, which may reflect either general or specific PIT, enhancements have been observed (Derman & Lattal, 2024; Peciña et al., 2006; Pool et al., 2015) but also null effects (Pielock et al., 2013) and impairments (Derman & Lattal, 2022, 2024). In contrast, in tasks unambiguously measuring specific PIT, effects of stress are typically not observed (Derman & Lattal, 2022; Pritchard et al., 2018; Quail et al., 2017; Steins-Loeber et al., 2020; Vogel et al., 2018; although see Morgado et al., 2012); and there is only one report of stress specifically impairing general PIT (Quail et al., 2017), although the effect was mediated by responding to the *neutral* cue, and the sample size of 24 relatively small.

The effects of stress described above have been primarily on reward-related systems (Derman & Lattal, 2024)—and we are not aware of any studies examining the effects of stress on aversively motivated PIT. Given that stress is intrinsically aversive, the motivational nature of the task may well have an impact on the pattern of findings obtained, and so empirically speaking this seems like an important question to address. Accordingly, the purpose of the present experiments was to explore the effect of stress on an aversively motivated PIT task (cf. Nadler et al., 2011; Paredes-Olay et al., 2002), using the same design as Quail et al. (2017; cf. Nadler et al., 2011), so we could separate general and specific PIT. Based on previous findings we did not expect an effect of stress on specific PIT; but given the number of reports of effects in the ambiguous single-outcome task, and of an impairment in general PIT, we tentatively predicted an effect here.

## Experiment 1

We employed a version of the quasi-avoidance task used by Nadler et al. (2011; cf. Paredes-Olay et al., 2002). There is considerable evidence that PIT is observed in avoidance procedures as readily as in appetitive paradigms, in both animals (e.g., Campese et al., 2017; Hendersen et al., 1980; LoLordo, 1967) and humans (Gámez & Rosas, 2007; Lewis et al., 2013; Nadler et al., 2011; Paredes-Olay et al., 2002). Participants were told they were protecting themselves from three different types of attack while seeking treasure; thus, the attacks served as aversive outcomes (see Table 1). First, they underwent instrumental training in which each of two responses destroyed a specific type of attack (R1 ➔ no O1, R2➔ no O2); specifically, R1, for example, produced a picture of O1, with the message “*O1 destroyed.*” In the subsequent Pavlovian phase three stimuli were each paired with one of the three attacks (S1➔O1, S2➔O2, S3➔O3), and a fourth with no attack (S4➔nothing). Finally, in the transfer test, participants performed R1 and R2 in the absence of the outcomes while the four stimuli were presented. Greater performance of R1 than R2 during S1, and of R2 than R1 during S2, indicated specific PIT, while more performance of R1 and R2 during S3 than S4 was the measure of general PIT.

**Table 1:** Design of experiment

Group	STAI 1	Stress Induction Procedure (SIP)	STAI 2	Instrumental training	Pavlovian training	Pavlovian Assessment	PIT Test	STAI 3
Stress		Heavy metal music & aversive picture		R1→no O1	S1→O1	S1?	S1: R1, R2	
					S2→O2	S2?	S2: R1, R2	
				R2→no O2				
					S3→O3	S3?	S3: R1, R2	
Positive		Classical music & positive pictures			S4→nothing	S4?	S4: R1, R2	
Neutral		Café background noise & neutral pictures						

*Note.* S = Stimuli, O = Outcomes, R = Responses

To induce stress, we employed an online stress induction technique; to maximise the difference in stress among the various conditions we used three groups of participants. Participants in the *stress* condition were exposed to aversive images and heavy metal music, neutral group participants were shown pictures of neutral objects while listening to café background noise, and participants in the positive group looked at pictures of ‘cute’ animals and listened to calm classical music. To evaluate the effects of these stress induction procedures we used the State-Trait Anxiety Inventory (STAI; Spielberger et al., 1983). Although developed to measure state and trait anxiety, the state scale of the STAI is sensitive to psychological stress (Rule & Traver 1983), and state STAI scores covary with physiological measures of stress such as pulse rate (Sayette et al., 2001). Exploiting the possibility that the participants might associate the pictures with the sound experienced in this stage, we arranged for each group to experience the sound from their stress induction condition throughout PIT testing, to ensure the manipulation effects persisted throughout this phase. (See Supplementary Materials for further information.)

These experiments were based on the premise that ongoing stress enhances addictive behaviours, and so the stressor was imposed at the start of the experiment (cf. Derman & Lattal, 2022). However, any effect observed might be mediated by an effect on the test *or* on learning in the Pavlovian and/or instrumental stages (Stelly et al., 2020; Valenchon et al., 2017). Given the simple nature of the to-be-learned associations, we judged it unlikely that an effect of stress on learning would be detected, but to explore this possibility a Pavlovian assessment stage was administered before the test to ensure the Pavlovian associations had been learned learnt equally well in the different conditions (see Table 1).^1^

## Method

### Participants

A power analysis was conducted using G*Power version 3.1.9.6 (Faul et al., 2007) to determine the required minimum sample size. The results indicated the minimum total sample size was 42 to achieve a power of 0.8 for detecting a medium effect size *f* = 0.25, at a significance criterion of *α* =.05, for ANOVA (repeated measures, within–between interaction). All the participants were recruited via the Prolific online platform. Participants were adults with normal or corrected-to-normal vision with undergraduate or graduate degrees. In total, 72 adults completed this study (24 in each group). The stress group comprised 13 men and 11 women (mean age = 26.33, *SD* = 4.51, range: 21–37), the positive group 12 men and 12 women, (mean age = 24.88, *SD* = 3.73, range: 21–36), and the neutral group 15 men, and nine women (mean age = 25.38, *SD* = 3.06, range: 22–33). Gender and age did not differ systematically by group (*p*s >.6).

### Materials

#### Stress induction procedure

##### Pictures

**Fig. 1:**
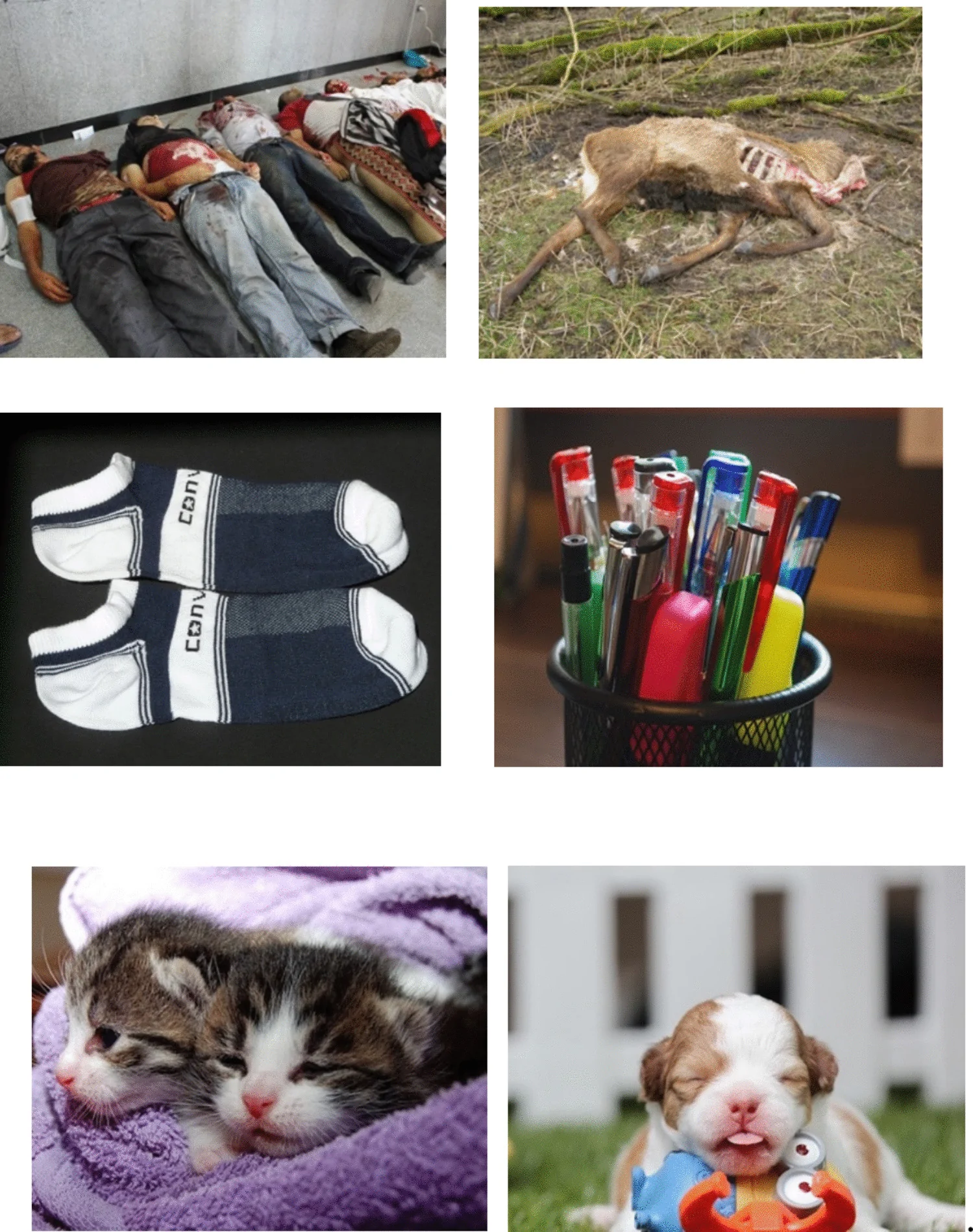
Examples of stress induction pictures for the three groups of Experiment 1. Top panel: stress condition; Centre panel: neutral condition; Bottom panel: positive condition. (Colour figure online)

The 12 negative, 12 neutral and 12 positive pictures were selected from the Open Affective Standardized Image Set (OASIS; Kurdi et al., 2017; see Supplementary Materials for further information); some examples from each condition are shown in Fig. 1. The mean arousal levels were 4.58, 2.12, and 4.00 for the negative, neutral and positive group respectively, where higher scores indicate higher arousal levels; the corresponding values for valence were 1.75, 4.19, and 5.89, where high values indicate positive valence.

#### Sounds

In the *stress* group, a 98-second clip from a heavy metal instrumental piece called ‘Fire’ was used (copyright-free), which was played repeatedly in a 144-second loop. A recording of café background noise was used as a neutral sound, and an excerpt from *Canon in D* (by Johann Pachelbel) for the positive condition.

#### Questionnaires

To measure stress, we used state anxiety questions from the STAI (Spielberger, 1970). Following Sayette et al. (2001), we used the short version (six items) of the STAI; such six-item short forms of the STAI have been shown to correlate highly with the full 40-item STAI (Marteau & Bekker, 1992; Tluczek et al., 2009). The STAI was presented, at the beginning of the experiment, immediately after the stress induction, and at the end of the experiment. The six items were:

1. *I am upset*
2. *I am worried*
3. *I am frightened*
4. *I am calm*
5. *I am secure*
6. *I am self-confident*

Participants clicked on a scale with the words ‘not at all’, ‘somewhat’, ‘moderately so’, ‘very much so’ to indicate how they felt at the moment for each statement. The answers were scored from 1 to 4, where a higher score indicated stronger feelings of anxiety (so items 4–6 were reverse-marked). The STAI score was the sum of the scores for each question.

#### PIT

**Fig. 2:**
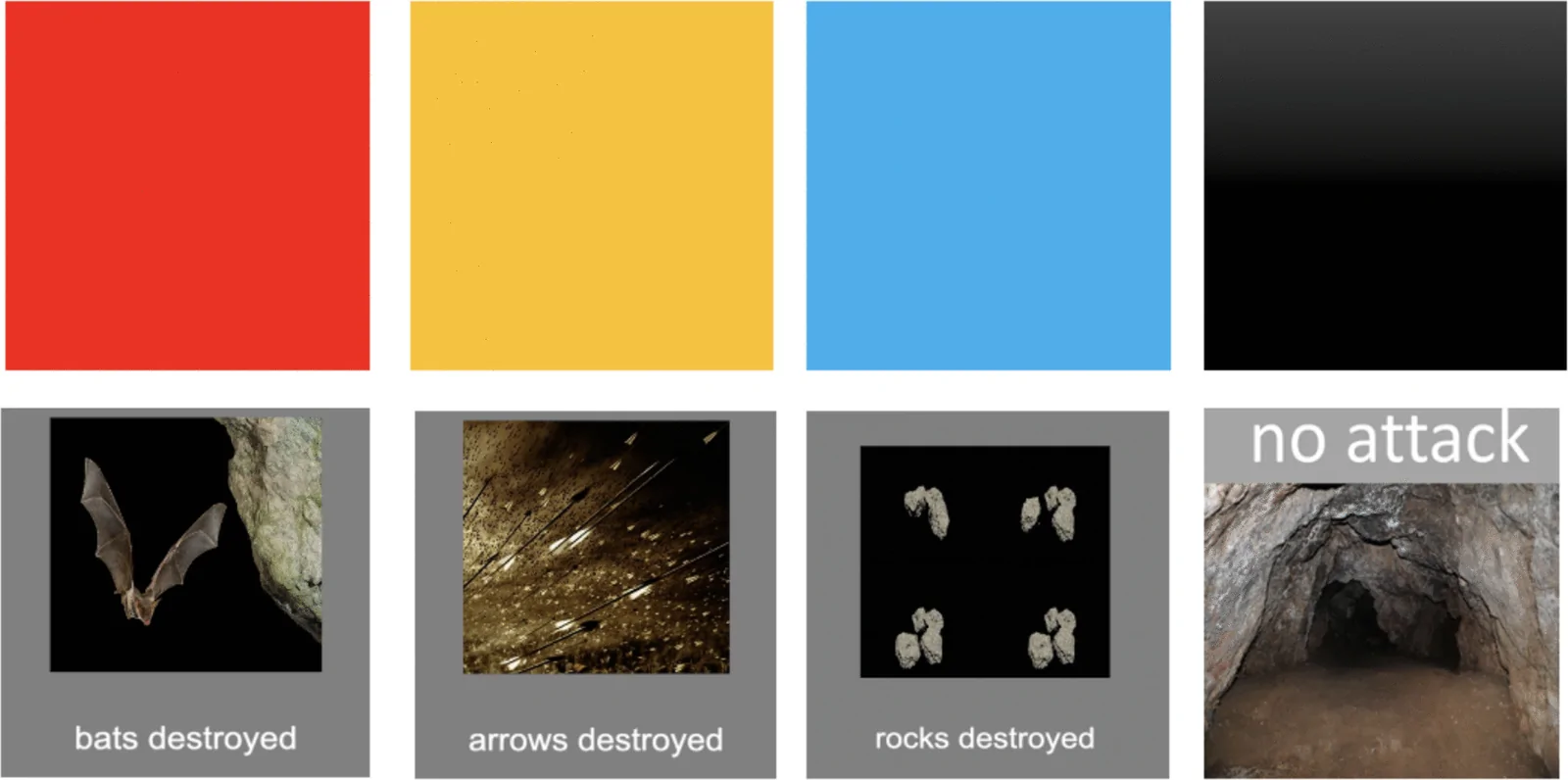
Stimuli (**top panel**) and instrumental outcomes (**bottom panel**) from the PIT task of Experiment 1. (Colour figure online)

**Fig. 3:**
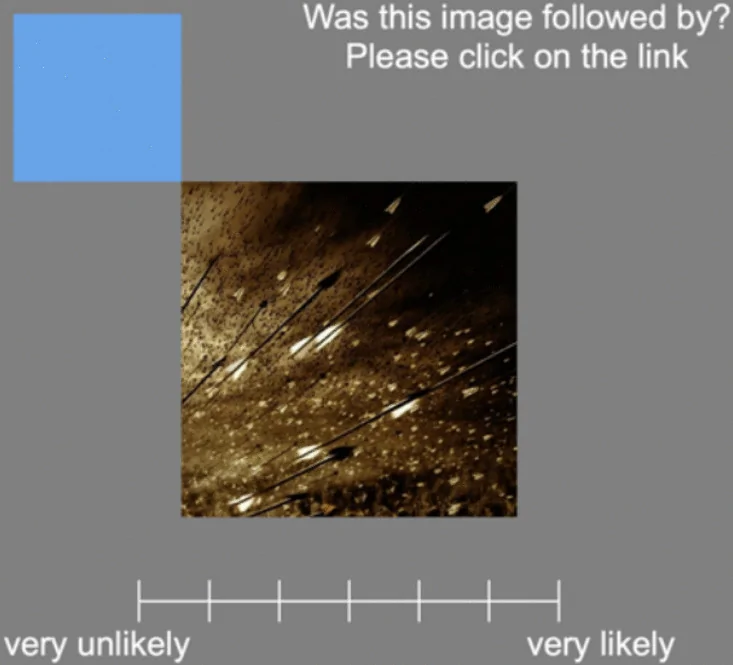
Example of question from the Pavlovian assessment of Experiment 1. (Colour figure online)

The PIT task was conducted using PsychoPy software (Peirce, 2007) and conducted online at Pavlovia.org. Participants completed the task on their own devices (laptop or computer). Responses were pressing the ‘z’ and ‘m’ keys on the keyboard. Stimuli (S1, S2, S3, S4) were red, yellow, blue and black coloured squares, and the outcomes pictures of the types of attack—bats, rocks and arrows; a picture of an empty cave with the word ‘no attack’ served as the non-reinforcement outcome (see Fig. 2). The identity of the outcomes paired with the *z* and *m* responses was counterbalanced, giving three counterbalancing conditions; within each the identities of S1, S2, S3, and S4 were also fully counterbalanced, giving a total of 24 counterbalancing conditions (see Supplementary materials). Both stimulus and outcome images were approximately 60 mm × 60 mm and presented in the centre of the screen, except in the Pavlovian assessment where the stimuli were arranged as in Fig. 3, and the question of ‘Was this image followed by? Please click on the link’ was presented at the top of the screen, and a rating scale from 1 = ‘very unlikely’ to 7 = ‘very likely’ at the bottom (see Fig. 3). Full instructions are presented in the Supplementary Materials.

### Procedure

The experiment received ethical approval (No. S1222) from the University of Nottingham School of Psychology Ethics Committee.

#### Questionnaires–STAI I

The six questions were presented sequentially on the screen, with the response scale. Participants responded by clicking on the scale, and the next question was presented after an intertrial interval (ITI) of 1 s, meaning the whole stage could take as little as 15 s.

#### PIT–cover story

At the start of the PIT task, participants were told they had to look for treasure, but that they would be subject to attacks from arrows, bats and falling rocks, against which they could defend themselves with a shield, a torch and a helmet.

#### Stress induction procedure

Participants were told that they would see images and sounds. They were reminded that if they were upset by any of the images they were free to withdraw from the experiment by closing the computer window and aborting the experiment; no participants withdrew from the study. To check if participants could hear the sound and as an attention check, they were then asked:

*‘What kind of sound you can hear?*

*click on the scale’*

Participants could choose ‘*a hubbub of voices*’, ‘*heavy metal music*’ or ‘*classical music’*.

In each trial in this procedure, a fixation cross was presented in the centre of the screen for 1 s, followed by a picture presented in the centre of the screen for 3 s. The 12 pictures were repeated three times in a semi-random order, giving a total of 36 trials. During the whole procedure, the heavy metal music/café noise/classical music was on. This stage lasted 144 s (2.4 min).

#### Questionnaires–STAI II

As STAI I.

#### Instrumental training

In the instrumental stage participants were told that they could avoid the various attacks by pressing the ‘z’ key and ‘m’ keys (Fig. 2), and were encouraged to protect themselves. The instrumental outcomes were delivered according to a concurrent variable ratio VR5–VR5 schedule (i.e., on average one in five responses was reinforced). R1 was followed by a picture of O1 (e.g., bats) with the words ‘*bats destroyed*’ below, while R2 was followed by a picture of O2 (e.g., arrows) and the words ‘arrows destroyed’ below. Each outcome was presented for 1 s. This stage finished after participants had earned at least 50 O1 and 50 O2. The duration of this stage depended on the speed of participants’ responding, but could take as little as 2 min. No outcomes were presented in the absence of a response.

#### Pavlovian conditioning

In the Pavlovian phase the stimuli were followed by pictures of the attacks (see Table 1). Participants were told they would receive signals indicating the upcoming attack, and asked to passively observe the relationships between the four stimuli and the four attacks. S1 and S2 were respectively paired with O1 and O2, the attacks used in the instrumental stage, while S3 was paired with the remaining attack O3, and S4 with the *no* *attack* image. Each stimulus/attack pairing was presented once in a semi-random order in each block, giving four trials per block. In each trial participants were presented with a fixation cross for 1 s, after which the stimulus was presented for 2 s, followed by the outcome picture for a further 2 s. There were 10 blocks in total, giving a total of 40 trials with a total duration of 200 s.

#### Pavlovian assessment

This was to check learning in the Pavlovian stage was equivalent in the three groups. Participants were told:

*‘Now you will be tested on your knowledge about the different signals. Use the mouse to click on the line to indicate confidence in your answer.’*

One of the four stimuli was presented with one of the four attacks (see Fig. 3). Participants needed to indicate if the two pictures matched by clicking on the scale at the bottom of the screen. Once the participant had made their rating, the next question appeared after an ITI of 1 s. Each of the four stimuli was presented with each of the four attacks once, giving 16 stimulus–attack pairs presented in a semi-random order. If participants took 1 s to make each rating, this stage would have lasted 32 s.

#### PIT test

In the PIT test participants continued to defend themselves by pressing the keys while the four Pavlovian conditioned stimuli were presented. The heavy metal music/café noise/classical music was on throughout this stage. Following the procedure employed by Alarcón and Bonardi (2016), each trial comprised a stimulus presented for 2 s, preceded by a 2-s pre-CS period and followed by a 2-s intertrial interval (ITI). The four different stimuli were presented once per block in a semi-random order, and there were six blocks of testing (24 trials), which lasted 144 s (2.4 min).

#### Questionnaires–STAI III

As STAI I.

Debrief documents were provided on request.

### Data treatment

The intention was to exclude any participants who incorrectly reported what type of music they had heard in the stress induction stage, but no participant failed this criterion. In total, data from 72 participants (24 in each group) was used for analysis.

In the Pavlovian assessment we did not measure accuracy directly, but participants’ ratings of their confidence that the outcome would be presented were recorded for each of the 16 stimulus/outcome combinations, from zero (low confidence) to 7 (high confidence). Then a mean rating was derived for each participant, for both correct and incorrect combinations. Our assumption was that, if they had learned that stimulus/outcome combination, they should show higher confidence for correct than incorrect pairings.

PIT data are presented pooled over the first three blocks of the test, as on the basis of our previous work we anticipated that any effects would be transient (cf. Alarcón & Bonardi, 2016; in fact, including all six test blocks did not affect the pattern of results observed; see Supplementary Materials where the data from all six blocks are analysed). *Z* and *M* responses during each of the four trial types (S1, S2, S3 and S4) were separated according to whether they occurred during the *preCS* periods (the 2-s periods preceding CS presentation) or the 2-s *CS* presentations. Response rates during the preCS and CS periods (in responses per minute [rpm]) were then computed for four conditions: congruent, incongruent, S3, and S4. Congruent referred to the response that had signalled the same outcome as that predicted by the stimulus (R1 in S1, R2 in S2), and incongruent to the alternative response (R1 in S2, R2 in S1). The S3 and S4 conditions were the total numbers of responses (R1+R2) on S3 and S4 trials respectively. Finally, difference scores—computed by subtracting response rates in the preCS period from those during the CS period—were calculated as a measure of the effect of CS presentation on background responding. The comparison between congruent and incongruent difference scores was the measure of specific PIT, and that between S3 and S4 difference scores the measure of general PIT. The resulting difference scores were subjected to an analysis of variance (ANOVA), with group and trial type (congruent versus incongruent, or S3 versus S4) as factors. The significance level was set at *p* <.05. Significant interactions were explored using simple main effects analysis, with the pooled error term; *η*_*p*_^*2*^ was reported for significant effects and interactions. The same analyses were performed on the preCS scores to rule out the possibility that differences in preCS responding were driving differences in the difference scores. In addition, we used a Bayesian ANOVA to assist interpretation of the key null results. In hypothesis testing, Bayes factors can serve as a quantitative measure of the predictive performance of the data relative to the null model. We report BF_01_; values of BF_01_ between 3 and 10 constitute *some* evidence for the null model over alternatives, and values over 10 *strong* evidence (cf. Jeffreys, 1961, cited in Rouder et al., 2009). Statistical analyses were performed in SPSS and JASP (Version 0.18.3; JASP Team, 2024).

### Results

#### Stress induction

**Fig. 4:**
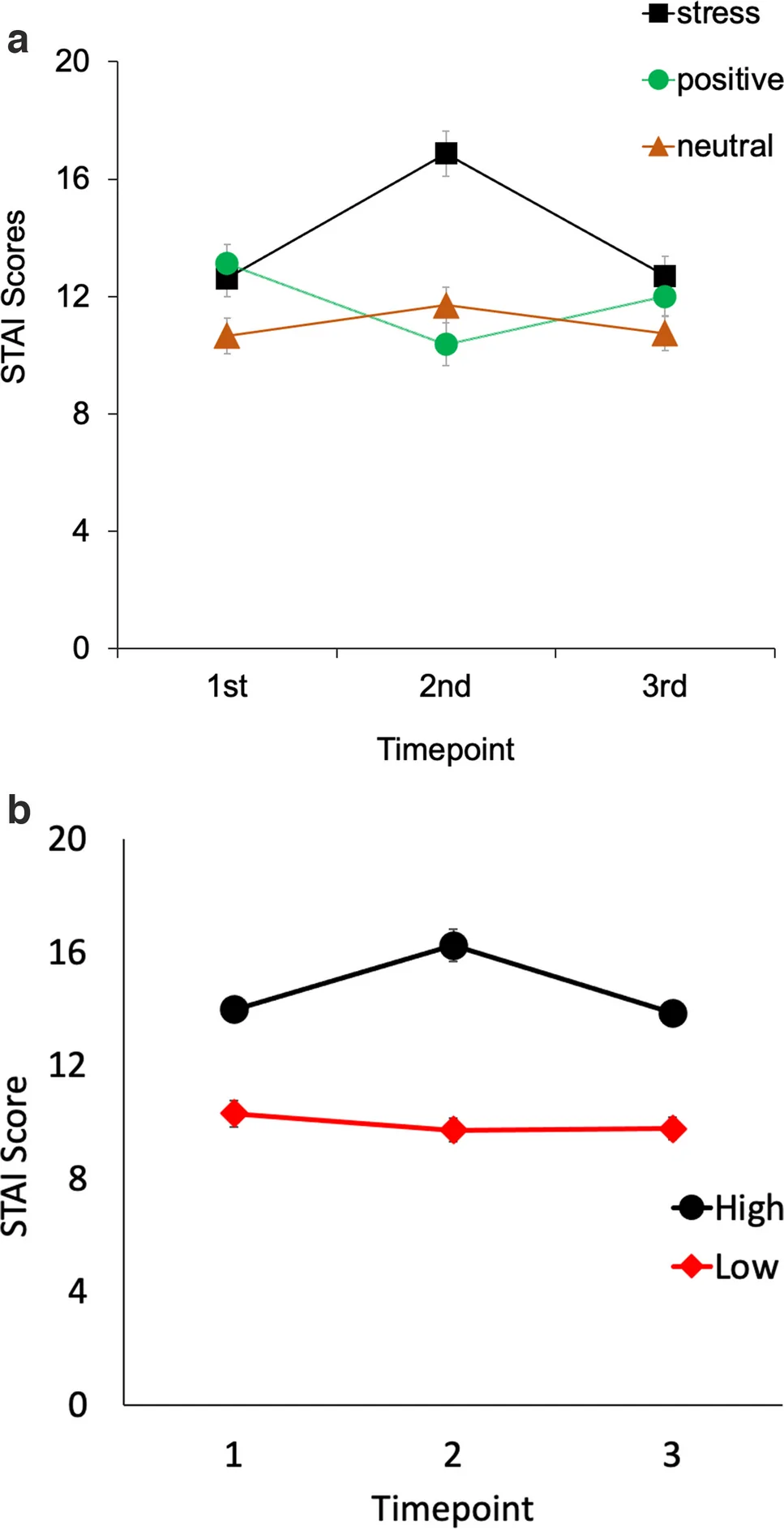
**a** Group mean STAI scores for the Stress, Positive and Neutral groups at the three time points (baseline, post-intervention and post-PIT) of Experiment 1. Error bars show standard errors of the mean. **b** Group mean STAI scores for the High and Low groups at the three time points (baseline, post-intervention and post-PIT) of Experiment 1. Error bars show standard errors of the mean

Figure 4a below illustrated participants’ scores on the STAI. Participants’ scores seemed to be similar at the beginning and end of the experiment, but participants in the stress group reported higher scores immediately after the induction procedure than those in the positive or neutral groups. This impression was supported by a Group × Time Points ANOVA, which showed a significant main effect of groups,* F*(2,69) = 8.812, *p* <.001, *MSE* = 20.148, ${\eta }_{P}^{2}$ =.203 and time points, *F*(2,138) = 4.55, *p* =.012, *MSE* = 5.754, ${\eta }_{P}^{2}$ =.062, and a significant interaction, *F*(4,138) = 14.726, *p* <.001, *MSE* = 5.754, ${\eta }_{P}^{2}$ =.299. Simple effects analysis revealed that the group effect was only significant on the second time point, *p* <.001; post hoc Tukey tests found scores were higher for the stress group than the neutral or positive groups (*p*s <.001), which did not differ, *p* =.89. This suggests the stress induction procedure was effective in enhancing stress, but this effect did not persist until the end of the experiment.

In view of the lack of sustained effects of the stress manipulations of the STAI scores, we dropped the group allocations and instead ranked participants on the basis of their average STAI scores across the three tests. We then created a high-stress group (comprising 19, nine, and eight participants from the stress, positive, and neutral groups, respectively) and a low-stress group (comprising five, 15, and 16 participants from the stress, positive and neutral groups). Their data are shown in Fig. 4b; an ANOVA with group and time points as factors revealed a main effect of group, *F*(1,70) = 163.98, *p* <.001, *MSE* = 7.459, ${\eta }_{P}^{2}$ =.701 and time points, *F*(2,140) = 3.50, *p* =.033, *MSE* = 7.478, ${\eta }_{P}^{2}$ =.048 and a significant interaction, *F*(2,140) = 5.75, *p* <.004, *MSE* = 7.478, ${\eta }_{P}^{2}$ =.076; simple main effects confirmed that the groups differed on all time points, *p*s <.001.

#### Pavlovian assessment

The group mean scores for correct and incorrect combinations were 6.31 and 1.32 for Group High and 6.43 and 1.34 for Group Low. ANOVA with trial type and group as factors revealed a significant effect of trial type, *F*(1,70) = 613.98, *p* <.001, *MSE* = 1.49, ${\eta }_{P}^{2}$ =.89, but no effect of group or interaction, *F*s < 1. The Bayes factors BF_01_ associated with the effects of group and the interaction were 5.05 and 3.97, respectively. As a further check we computed ratio scores of form correct/(correct + incorrect), where higher ratios indicate better performance. The mean ratios were 0.871 and.821 for Groups High and Low, respectively, and one-way ANOVA confirmed that these scores did not differ, *F* < 1 (BF_01_ = 4.08).

#### Specific PIT

**Fig. 5:**
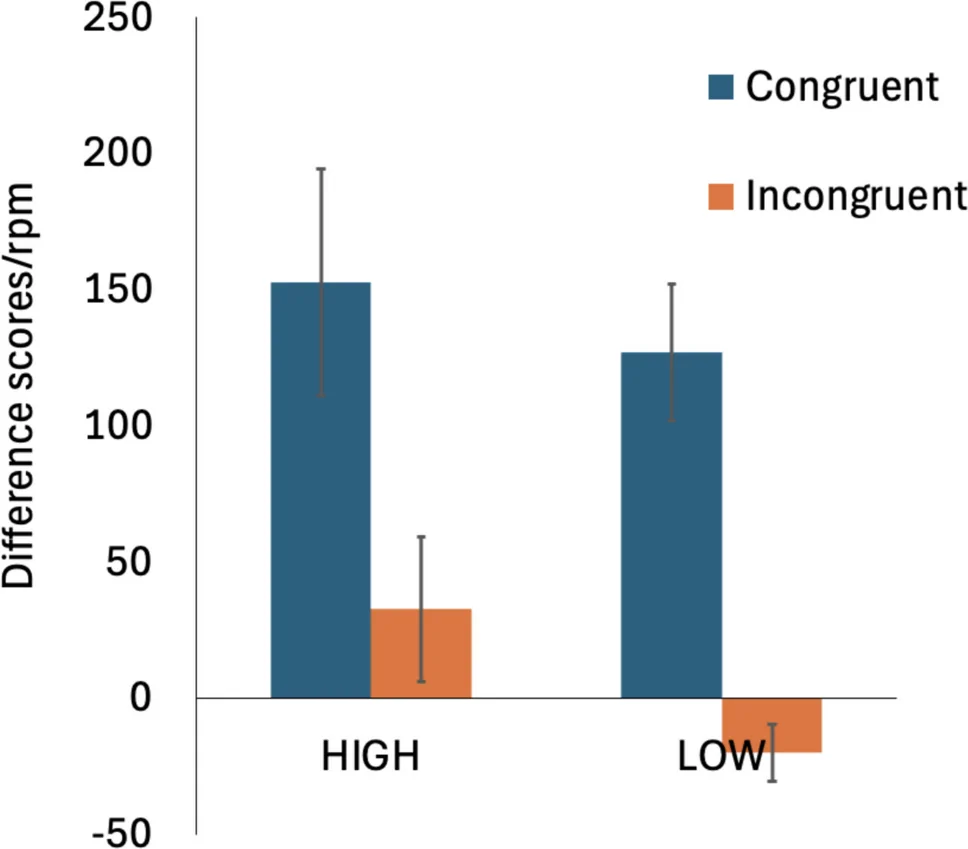
Group mean difference scores for congruent and incongruent conditions of the specific PIT test pooled over the first three test blocks of Experiment 1. Error bars show the standard error of the mean

The CS-preCS scores for the congruent and incongruent conditions are presented in Fig. 5. Congruent scores appeared higher than incongruent in both groups, and there was little evidence of a difference between the groups; ANOVA with group and condition (Congruent, Incongruent) as factors revealed a main effect of condition, *F*(1, 70) = 18.12, *MSe* = 106058, *p* <.001,η_p_^2^ =.206; neither the main effect of group or the interaction were significant, *F*(1, 70) = 2.51, *MSe* = 65747, *p* =.11, and *F* < 1, respectively. Nonetheless the BF_01_ associated with the group × condition interaction was 6.05, providing some evidence for the null hypothesis.

The mean preCS rates for congruent and incongruent trials were respectively 112.78 and 89.72 rpm for Group High, and 45 rpm and 68.89 for Group Low. An ANOVA with group and condition as factors found no difference between these scores, largest, *F*(1, 70) = 1.61, *MSe* = 12301, *p* =.21, for the interaction.

#### General PIT test

**Fig. 6:**
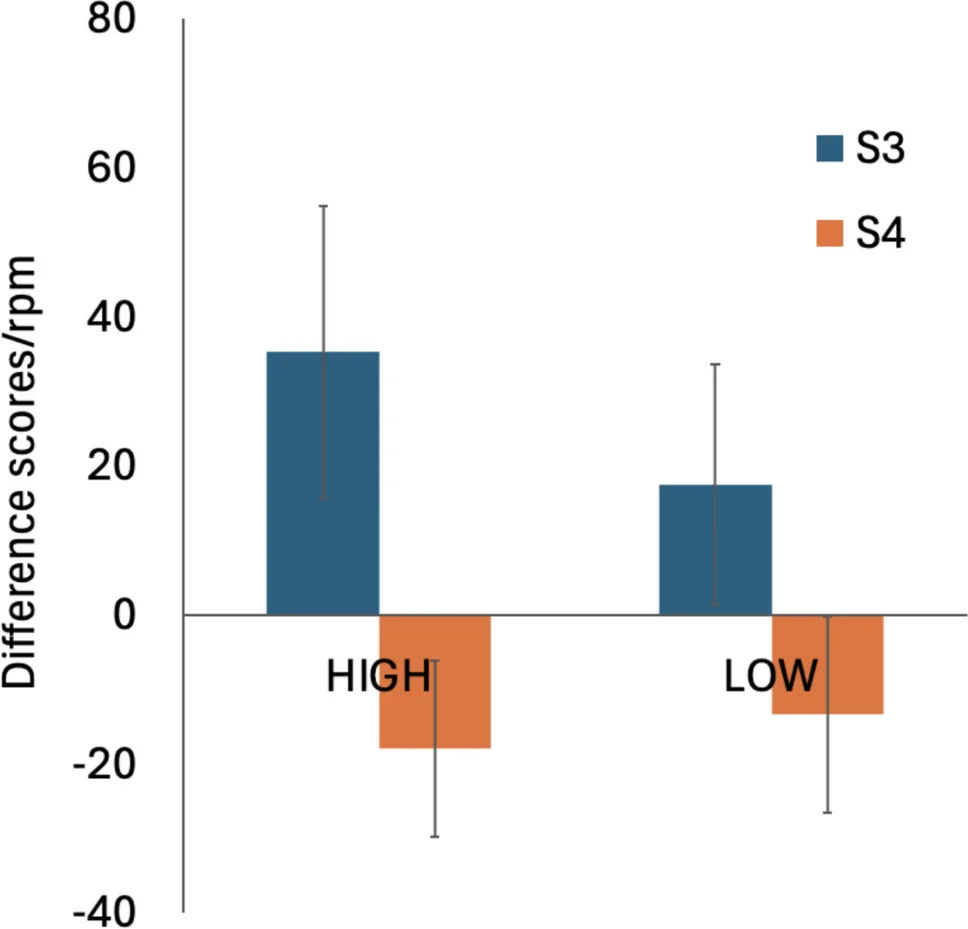
Group mean difference scores for S3 and S4 of the general PIT test pooled over the first three test blocks of Experiment 1. Error bars show the standard error of the mean

Difference scores for the S3 and S4 conditions are presented in Fig. 6. Scores for S3 were greater than for S4 in both groups, and this difference appeared slightly greater in the High condition—but an ANOVA with group and condition (S3, S4) as factors revealed only a main effect of condition, *F*(1, 70) = 9.38, *MSe* = 6772, *p* =.003, η_p_^2^ =.118; neither the main effect of group nor the interaction were significant, *F*s < 1. Here stress had no clear influence on the size of the general PIT effect, although the Bayesian analysis yielded BF_01_ for the interaction of 2.84, some evidence in favour of the null model.

The preCS rates were, respectively for S3 and S4 trials, 72.91 and 102.92 rpm for Group High, and 47.08 and 58.61 rpm for Group Low; a Group × Trial Type ANOVA revealed nothing significant, largest *F*(1,70) = 3.25, *MSe* = 4774, *p* =.076, η_p_^2^ =.044, for the main effect of condition.

### Discussion

Both specific and general effects were clearly evident in the PIT test, in line with previous work showing both specific and general PIT with humans in avoidance-based PIT tasks (Lewis et al., 2013; Nadler, et al., 2011). However, there was no evidence that either general or specific PIT differed between the two groups. This was despite a fairly substantial difference in STAI scores between the two conditions (with overall mean scores of 14.69 [1.60] and 9.94 [1.55] in the high and low conditions, respectively, standard deviations shown in brackets). This group difference in STAI score of 4.75 was substantially larger than the difference of 2.1 produced by the stress manipulation in the Sayette et al. (2001) study, and which was accompanied by a significant increase in pulse rate. Although this difference between the High and Low groups in their state anxiety scores was clearly not a result of the experimental manipulations, it could still have stemmed from random differences in state stress among the participants.

The relative ineffectiveness of the stress induction procedure (SIP) deserves comment. Although the SIP elevated STAI scores in the stress group relative to the other conditions, this difference did not persist until the end of the task. It is unclear why the stress manipulation’s effect was not more long-lasting—but it might be because it was conducted online. Participants were thus likely to be taking part in a familiar environment of their own choosing, limiting the capacity of our procedure to affect stress levels in a durable manner. Alternatively, the 6-minute interval between the stress induction procedure and the PIT test might have been long enough for any effects to abate. Moreover, we did not ask participants about their music preferences—people who like heavy metal music might have found it less stressful than those who do not; alternatively, it may be that the music distracted them from attending to the pictures. To address these factors, Experiment 2 employed an in-person stress induction procedure, and the whole experiment was conducted in the laboratory.

## Experiment 2

In Experiment 2 a speech stress induction procedure, modified from one used by Sayette et al. (2001), was employed to try and ensure the effects would be more profound and long-lasting. Participants in the *stress* group were told that they would have to give a speech about what they liked and disliked about their bodies at the end of the experiment, and that the speech would be filmed and later analysed by psychologists. A strong photographic light was on throughout the study, allegedly for the filming later. In addition to adding credence to the cover story, light conditions can influence humans’ stress levels: for example, it has been reported that adults exposed to bright light had higher stress hormone levels than others exposed to dim white light (Petrowski et al., 2020). There was also a *neutral* group, in which participants were asked to read magazines, and for whom the experiment was conducted in normal lighting conditions, and a *positive* group, who was asked to practice a brief relaxation technique, and completed the whole experiment under dim light. The latter group’s treatment was based on evidence that short relaxation practice can significantly decrease heart rate and blood pressure (Pal et al., 2014). In all other respects this experiment was identical to Experiment 1, except that there was a short reminder of the stress induction procedure just before the transfer test (see Table 2).

**Table 2:** Design of Experiment 2

Group	STAI 1	Stress Induction Procedure	STAI 2	Instrumental training	Pavlovian training	Pavlovian Assessment	Stress Induction Reminder	PIT Test	STAI 3
Stress		Speech Stressor Task		R1→no O1	S1→O1	S1?		S1: R1, R2	
		Bright light		R2→no O2	S2→O2	S2?		S2: R1, R2	
Neutral		Read Magazines Normal light			S3→O3	S3?		S3: R1, R2	
Positive		Relaxation exercise Dim light			S4→nothing	S4?		S4: R1, R2	

*Note.* S = Stimuli, O = Outcomes, R = Responses

### Method

Unless specified otherwise procedures were identical to those of Experiment 1.

#### Participants

Seventy-two students studying at the University of Nottingham completed this study (24 in each group), with a mean age of 22.45 (*SD* = 4.09, range: 18–35). In the *stress* group, there were six men and 17 women, and one who did not report gender; the mean age was 20.92 years (*SD* = 3.19, range: 18–29). In the *neutral* group, there were nine men and 15 women, with a mean age of 22.54 years (*SD* = 4.06), range: 19–35). In the *positive* group there were 12 men and 12 women; the mean age was 23.79 years (*SD* = 4.55, range: 18–33). Gender and age did not differ systematically by group (*p*s >.1). No participants withdrew from the experiment.

### Materials

#### Stress induction

##### Speech stressor task

A Panasonic GS250 Camcorder, a camera tripod, and a Vivanco videoleuchte (300 Watt) were used in the stress group. The magazine used in the neutral group was ‘National Geographic–Lost cities’ from November 2021. In the positive group a small night light (0.5-watt) and a 5-min piece of calm music with guided relaxation practice were used: participants were told to sit comfortably, clear their thoughts and focus on their breath, breathe in and out slowly to relax, and listen to some positive phrases (e.g., you will be confident).

### Procedure

#### Stress Induction 1

A modified version of the speech stressor task conducted by Sayette et al. (2001) was applied in the stress group. The camera, with the photographic light beside it, was placed in a corner of the experimental room. A seat was placed two meters away, facing the camera, with a white wall behind it as background. At the beginning of the experiment, participants were shown into the room and told they would have to give a speech about what they liked and disliked about their physical appearance, that they should be as honest as possible, and that a therapist would rate it for defensiveness (see Supplementary Materials).

This procedure took about five minutes, and both the room light and the photographic light were on; these lighting conditions were maintained for the rest of the task in this group.

Participants in the neutral group were simply asked to read the magazine in the experimental room for 5 min, under the normal room light, which was kept on for the rest of the experiment. Participants in the positive group were asked to follow the relaxation practice instructions for 5 min; for this group the whole experiment took place in the dim light.

#### Stress induction reminder

Just before the start of the PIT test one sentence was presented on the screen as a reminder of the stress induction procedure—‘*Reminder: you will give the speech in about five minutes…’* for the stress group, ‘*Reminder: you will read the magazine in about five minutes...*’ for the neutral group and ‘*Relax.. *.’ for the positive group. After reading the sentence, participants were invited to press ‘Space’ to begin the PIT test. As noted above, the lighting conditions each group experienced during the stress induction persisted throughout PIT testing.

Finally, the participants were debriefed and thanked for their participation.

### Results

The data treatment procedure was the same as in Experiment 1.

#### Stress induction

**Fig. 7:**
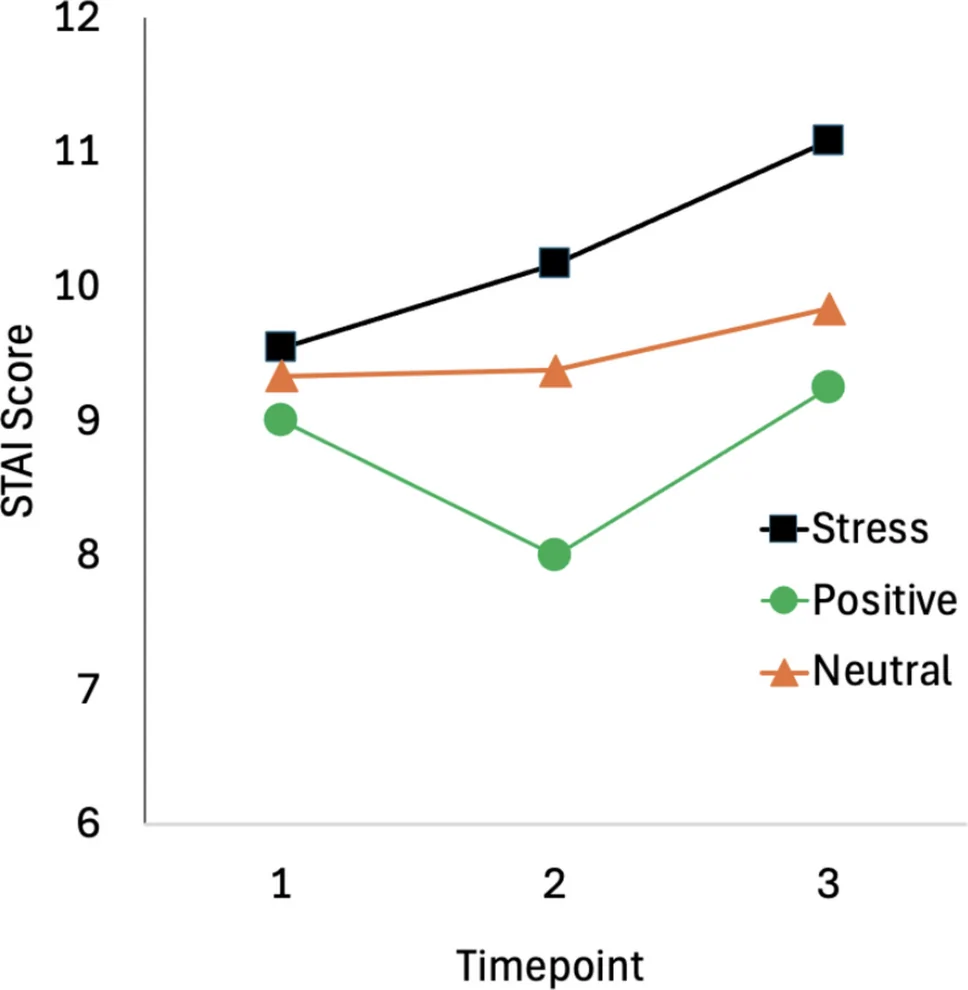
Group mean STAI scores at the three time points (baseline, post-intervention and post-PIT) of Experiment 2. Error bars represent standard errors of the mean

The STAI scores for the three groups (Stress, Positive, Neutral) at the three different time points of the experiment are shown in Fig. 7. The scores from the stress group appeared to increase over the course of the experiment, while those from the neutral group remained steady; scores from Group Positive were lower than those of the other two groups. A Group (stress, positive, neutral) × Time Points mixed ANOVA revealed significant main effects of groups,* F*(2,69) = 3.169, *p* =.048, *MSE* = 13.017, ${\eta }_{P}^{2}$ =.084, and of time points, *F*(2,138) = 5.968, *p* =.003, *MSE* = 2.738, ${\eta }_{P}^{2}$ =.080; the interaction was not significant, *F*(4,138) = 1.907, *p* =.113, *MSE* = 2.738. Tukey’s test revealed that the stress group differed from the positive group, *p* =.037, but none of the other group differences attained significance, smallest *p* =.43.

As our main concern was whether the manipulations successfully affected stress, we conducted a further analysis omitting the neutral group. This confirmed a significant effect of group,* F*(1,46) = 6.76, *p* =.012, *MSE* = 12.21, ${\eta }_{P}^{2}$ =.128, as well as timepoint,* F*(2,92) = 6.432, *p* =.002, *MSE* = 2.50, ${\eta }_{P}^{2}$ =.12 and a significant interaction,* F*(2,92) = 3.53, *p* =.033, *MSE* = 2.50, ${\eta }_{P}^{2}$ =.07; simple main effects confirmed that the groups differed at Timepoints 2 and 3, *p* <.001 and *p* =.027, respectively, but not at Timepoint 1,* p* =.41. Thus, we can conclude that either or both of the stress and positive conditions had an effect on stress that persisted until the end of the study. Thus, to magnify the differences between the stress conditions, we confined our remaining analyses to these two groups (see Supplementary Materials for analysis of the data from all three groups).

#### Pavlovian assessment

The group mean scores for correct and incorrect combinations were 6.52 and 1.50 for Group Stress, 6.36 and 1.51 for Group Positive. An ANOVA with trial type and group as factors revealed a significant effect of trial type, *F*(1,46) = 388.57, *p* <.001, *MSE* = 1.50, ${\eta }_{P}^{2}$ =.89, but no effect of group or interaction, *F*s < 1. The Bayes factors *BF*_*01*_ associated with group and the interaction were 4.32 and 3.13, some evidence for the null hypothesis. The mean ratio scores were 0.813 and 0.808 for Groups Stress and Positive, respectively; a one-way ANOVA confirmed that these scores did not differ, *F* < 1 (BF_01_ = 3.42). Again, there was no evidence that the groups differed in their ability to learn the Pavlovian associations.

#### Specific PIT

**Fig. 8:**
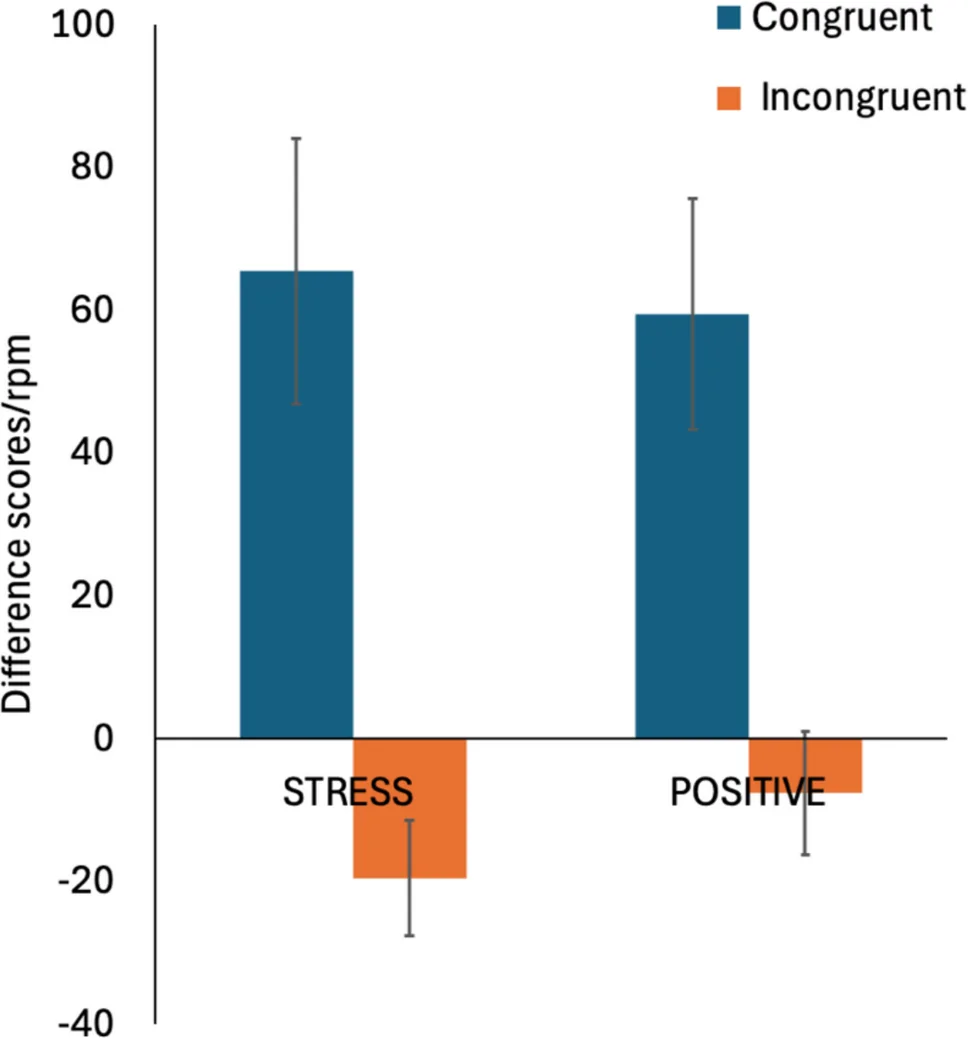
Group mean difference scores for congruent and incongruent conditions of the specific PIT test pooled over the first three test blocks of Experiment 2. Error bars show the standard error of the mean

The Difference scores for the congruent and incongruent conditions are presented in Fig. 8. Once again there was a clear effect of congruency but little indication of a group difference: an ANOVA with group and condition as factors revealed a main effect of condition,* F*(1,46) = 35.67, *p* <.001, *MSE* = 3618, ${\eta }_{P}^{2}$ =.44, but nothing else was significant, *Fs* < 1. BF_01_ for the effect of group was 3.91, and 3.48 for the interaction, providing some evidence in favour of the null model.

The mean preCS scores for congruent and incongruent trials were 50.83 and 61.25 rpm for Group Stress and 59.38 and 54.38 rpm for Group Positive. An ANOVA with group and condition as factors confirmed that these scores did not differ, largest *F*(1,46) = 2.05, *p* =.16, *MSE* = 695, for the interaction.

#### General PIT

**Fig. 9:**
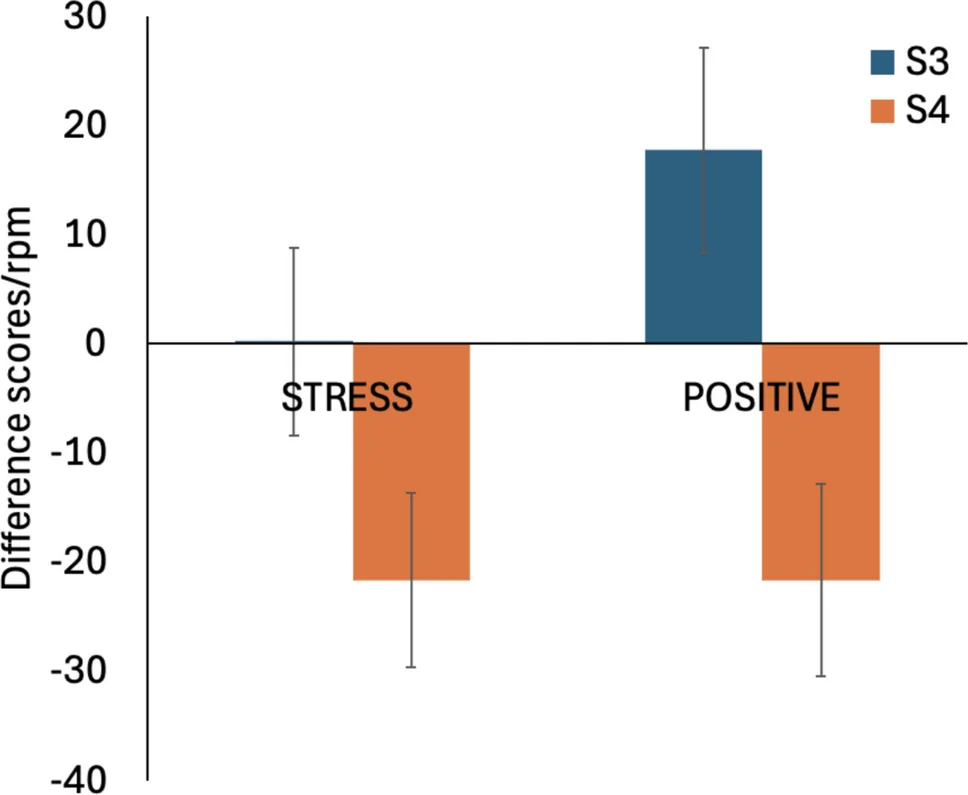
Group mean difference scores for S3 and S4 of the general PIT test pooled over the first three test blocks of Experiment 2. Error bars show the standard error of the mean

The difference scores for the S3 and S4 conditions are presented in Fig. 9; this suggests that rates during S3 were higher than those during S4; in contrast to the previous experiment, where the high group showed the numerically higher general PIT effect, here it seemed to be smaller. Nonetheless, ANOVA with group and condition as factors revealed a main effect of condition,* F*(1,46) = 17.41, *p* <.001, *MSE* = 1293, ${\eta }_{P}^{2}$ =.28, but nothing else significant, largest *F*(1,46) = 1.42 *p* =.24, *MSE* = 1295 for the interaction (with BF_01_ = values of 2.86 and 1.99 for the effects of group and the interaction respectively). The mean preCS scores for congruent and incongruent trials were 55.00 and 57.71 rpm for Group Stress and 56.04 and 57.91 rpm for Group Positive. An ANOVA with group and condition as factors confirmed that these scores did not differ, *F*s < 1.

### Discussion

In this experiment the stress and positive manipulations produced a clear difference in stress between the two groups which persisted until the end of the experiment. Nonetheless, we again failed to find an effect of stress on PIT: the specific and general PIT effects, although clear and reliable, did not differ between the two groups. Nor, as in the previous experiment, was there any discernible effect of the stress manipulation on the ability of participants to acquire the Pavlovian associations.

To bolster the conclusion that our stress manipulations did not influence PIT, and also take account of the fact that the manipulations we employed take no account of individual differences in baseline stress or susceptibility to the various stress manipulations, we exploited the fact that the same measure of stress was used in Experiments 1 and 2. Thus we combined the complete data set from the two studies (including Group Neutral from Experiment 2) and performed nonparametric (Kendall’s tau) Bayesian correlations between individual stress levels (pooled across the second and third timepoints) and the size of the two PIT effects. To correct for differences in absolute levels of responding, we computed ratio scores to reflect the size of the PIT effects. We pooled responses during the CS across the three test blocks for the congruent, incongruent, S3 and S4 conditions, and computed two PIT ratios, (congruent/(congruent + incongruent)) for specific PIT, and (S3/(S3 + S4)) for general PIT. Scores above 0.5 indicate more responding on congruent than incongruent, or on S3 than S4, trials.

*Specific PIT* The correlation between PIT ratio and STAI was not significant, Kendall’s tau = −.013. Adopting the alternative hypothesis that specific PIT and STAI scores would be correlated, and setting stretched beta width to 0.5 (as we did not expect a strong relationship) gave a Kendall’s tau of −.024; the Bayes factor suggested moderate evidence for the null hypothesis, BF_0+_ = 5.66.

#### General PIT

Response rates on S3 and S4 trials were routinely rather lower than on congruent and incongruent trials, with the result that 25 out of 144 participants (seven in the stress condition, 12 in the positive condition, and five in the neutral condition) made no responses in either preCS or CS period. This made computation of the ratio impossible (0/0) and so their data were omitted from the following analysis. Adopting the alternative hypothesis that specific PIT and STAI scores would be positively correlated, as outlined in the introduction, the correlation of the remaining PIT ratios and the STAI scores gave a Kendall’s tau of −.061; again, setting stretched beta width to 0.5, the Bayes factor indicated strong evidence for the null hypothesis, BF_0+_ = 10.55.

## General discussion

The aim of this study was to explore whether stress influences performance on specific and general avoidance-based PIT tasks in human participants—specifically, the empirically based suggestion that stress might affect performance on general, but not specific, PIT. However, we found no evidence for an effect of induced stress on either type of PIT. As far as we are aware this is the first study evaluating the effect of stress in such a quasi-avoidance-based PIT task. Whether this reflects a genuine absence of an effect of stress, or some more specific feature of our task or stress manipulation, is of course difficult to determine, and the Bayesian analyses we employed to evaluate support for the null hypothesis were not as conclusive as they might have been.

Our rationale for predicting a selective effect on general PIT was based on the failure of most studies to find an effect of stress on specific PIT, but the many reports of effects—both enhancements and impairments—in single-outcome tasks which could reflect both general and specific PIT, plus one report of a specific impairment in general PIT (Quail et al., 2017). There may also be theoretical reasons for expecting a selective effect on general PIT. The most standard explanation of PIT is in terms of associative learning. During the Pavlovian and instrumental stages, associations form between the various stimuli and responses and the outcomes (i.e. S1➔O1, S2➔O2, S3➔O3, S4➔nothing; R1➔O1, R2➔O2). The instrumental associations are postulated to work in reverse, such that presentation of O1 can elicit R1, and O2 R2 (Mackintosh & Dickinson, 1979; Pavlov, 1932). Outcomes are postulated to comprise *sensory* components (Konorski, 1967), referring to any nonaffective aspect that of the stimulus that differentiates one from another, and *affective* components, reflecting their motivational valence. Thus, specific PIT arises from an associative chain involving the *sensory* aspects of the outcomes: at test, S1 activates the sensory representation of O1 (which is not shared with O2), which selectively elicits R1. General PIT, on the other hand, arises from the ability of CSs signalling motivationally significant outcomes to elevate performance of responses motivated by outcomes of the same valence, via activation of a common motivational state (that in our study was shared by S1, S2, and S3, but not S4). These theoretical processes have been invoked to explain a wealth of addiction-related phenomena (Hogarth et al., 2013; although not everyone has managed to establish links between PIT and addiction; see, e.g., van Timmeren et al., 2020).

Potential effects of stress can be hypothesised within this theoretical framework. For appetitively motivated PIT, stress increases self-administration of addictive substances like heroin and cocaine in rats (Piazza & Le Moal, 1998; Shaham & Stewart, 1994; Will et al., 1998), and both stressors and corticosterone administration sensitise the dopaminergic response to cocaine (Rouge-Pont et al., 1995), suggesting that the effects on self-administration are mediated by enhancement of the drugs’ rewarding effects. If stress also magnifies the rewarding properties of the Pavlovian outcomes, under stress S3 would have greater motivational value, and be more effective at elevating responding than its nonreinforced counterpart S4. This account predicts enhanced general PIT—and as reviewed in the introduction, enhancements have indeed been observed (Derman & Lattal, 2024; Peciña et al., 2006, Pool et al., 2015). But impairments have also been seen: one marked example is the report by Derman and Lattal (2024), who found an enhancement in adult female rats but an impairment in adult males, using the same procedures (and interestingly, the impairment inmales was partially driven by elevated response rates to S4—the pattern of results observed by Quail et al., 2017). The authors suggested these findings may stem from sex differences in the effect of stress on cue-driven reward seeking (Derman & Lattal, 2024); they certainly highlight the complexity of the issue. Such an enhancement would not be predicted for specific PIT, where the key comparison is between the effects of two cues both associated with outcomes of enhanced motivational value, albeit different sensory properties.

A similar mechanism might be applied to aversively motivated PIT, by means of the *self-medication* hypothesis. Many have argued that addictions serve to reduce negative affect produced by external factors such as stress (Tomkins, 1966). For example, Tyssen et al. (1998) reported, in a large study of Norwegian students passing through medical school, a substantial association between hazardous drinking and using alcohol to deal with tension. According to this interpretation, indulging in addiction is effectively an avoidance response to escape or avoid the negative affect in everyday life. Stress, by heightening this negative affect, could make its removal more rewarding, thus elevating operant responding; but it could also increase the aversive potency of the CSs associated with the avoided situation—and cues signalling aversive outcomes facilitate aversively-motivated responding (cf. Rescorla & LoLordo, 1965; Rescorla & Solomon, 1967). Stress could therefore, just as in the appetitive case, enhance such *cue-driven* self-medication in general PIT—but again an effect with specific PIT would not be anticipated. This reasoning does not rule out the possibility that stress might interact with specific PIT in other ways, however. For example, some have argued that stress can bias behavioural strategies away from flexibility (Giovanniello et al., 2023) – although how exactly this might apply to specific PIT is beyond the scope of the present article.

Although our findings were relatively clear, we should acknowledge several limitations of the present work. The first relates to the measure of stress employed. In the present study we used the short version of the STAI which has been established to covary with pulse rate, a more orthodox measure of stress. But the other human studies have used different measures, and it is quite possible that one reason for the variability in the effects of stress on PIT in published work stems the wide variety of measures employed. In human tasks this can range from simply asking people to rate their stress level between 1 and 10, measuring salivary cortisol (Pool et al., 2015; Steins-Loeber et al., 2020), the Profile of Mood States (POMS; Pritchard et al., 2018), the Perceived Stress Questionnaire (PSQ20; Steins-Loeber et al., 2020), the Depression Anxiety Stress Scales (DASS; Quail et al., 2017), to the STAI in the present experiments. For example, it may be that stress has several underlying components that are not all captured by every test. Another problem with the use of multiple tests is that it allows no direct comparison of the degree of stress involved—which of course is likely to be a critical determinant of whether effects of stress are observed. The fact remains that we relied solely on self-reported measures of stress, and our conclusions would have been greatly strengthened by use of physiological measures. Future work would benefit from consistently using a range of stress indices, including physiological measures. Another issue relates to the levels of stress our manipulations were able to induce, which may simply not have been high enough to influence motivation—because, in common with many human studies, we used symbolic outcomes and so were unable to substantially manipulate motivational levels (cf. Nadler et al., 2011). In the absence of physiological measures of stress, it remains possible that our manipulations were more of mood than extant stress levels.

Our strategy of using symbolic outcomes rests on the assumption that the sensory and affective components proposed to comprise natural rewards can also be attributed to the pictures of the various attacks (Konorski, 1967)—corresponding respectively to the visual features of the different outcome pictures, and the affective reactions evoked by those images—imagining being bitten by bats, pierced by arrows or crushed by rocks. That tasks of this type engage true motivational processing is supported by Nadler et al.’s (2011) suggestion that they observed general PIT only when the task was adapted to give the outcomes greater motivational significance in the context of the task, by adopting a cover story, similar to that employed here. Our interpretation of our task was that the avoidance response was reinforced by omission of the motivationally negative outcomes. But other interpretations of the task are possible. First, as Nadler et al. (2011) noted, the event produced by the instrumental response—the attack accompanied by the message that the attack had been destroyed—is similar to the attack signalled by the Pavlovian CS, insofar as a salient feature of both outcomes is the attack itself. So, if the response is actually engaged in an *excitatory* association with the attack (as opposed to an inhibitory association signalling its absence), then PIT could be mediated just as it is in appetitive tasks. Moreover, we cannot rule out the possibility that participants were reasoning rationally about their behaviour, rather than relying on associative mechanisms. At present we are unable to discriminate among these possibilities. Finally, it should be noted that although participants’ responses were ostensibly followed by the absence of the attack, they did not experience pictures of the attacks if they did *not* respond; so, in this sense, our procedure was not a true avoidance task (hence, calling it quasi-avoidance).

Another factor to consider is the fact we administered our stress manipulation before the Pavlovian and instrumental training phases. This raises the possibility that an effect on PIT could have been mediated by an effect on learning rather than on test performance. For example, Derman and Lattal (2022, 2024) found evidence that the effect of their shock battery on general PIT in male rats was more reliable if it occurred before the Pavlovian and instrumental training phase; although they could not detect a reliable effect of shock on learning, numerically the shock battery appeared to attenuate learning in the training phase. But we found no evidence of variation in learning of the four Pavlovian associations in the different groups.

In summary, the study aimed to investigate the impact of stress on avoidance-based PIT tasks, hypothesizing that stress might affect general PIT performance. These experiments were designed to test one possible mechanism by which stress could affect addictive behaviours—namely, that this effect might be mediated by a direct effect of stress on PIT performance. We found no evidence for this assertion—our results do not support the notion that stress can affect PIT, either general or specific. But it may be that more sensitive or adequately powered experiments are necessary to conclusively determine the relationship between stress and PIT performance. This study is the only one of which we are aware that has explored the effect of stress on an aversively motivated task. Given the potential relevance of this mechanism to the self-medication components of addiction discussed above, it seems that further work is urgently needed to explore the effects of stress on tasks of this type.

## Supplementary Information

Below is the link to the electronic supplementary material.

## Data Availability

The raw data have been provided in the supplementary materials.

## Footnotes

1 Instrumental learning was not evaluated as it was anticipated that if participants had learned the four Pavlovian associations (each presented 10 times) they would also have retained the two instrumental associations, as these were each presented at least 50 times).

## Code availability

No specialised coding was required for this work.
