# Supplementary material for: Online and in-person stress induction in a quasi-avoidance-based Pavlovian-to-instrumental transfer task
Source: Learn Behav. 2026 Feb 12;54(3):302–18. doi: 10.3758/s13420-026-00707-5 (PMC13538191; doi:10.3758/s13420-026-00707-5)
Supplement: Supplementary file 1 [file 13420_2026_707_MOESM1_ESM.docx]

**EXPERIMENT 1**

**Stress Induction Procedures**

**Experiment 1** Participants in the *stress* condition were exposed to aversive images and heavy metal music; viewing pictures of dead bodies has been shown to significantly enhances stress levels (Pritchard et al., 2018), while listening to heavy metal music is less effective at reducing the negative emotion and arousal produced by a stressful event than classical or relaxing music (Labbé et al., 2007). Neutral group participants were shown pictures of neutral objects while listening to café background noise, and participants in the positive group looked at pictures of 'cute' animals and listened to calm classical music.

STAI was developed to measure state and trait anxiety. While trait anxiety is a type of individual difference, state anxiety is said to vary with situational stress: perception of threat produced by stressors in the environment increases state anxiety, which can lead to the physiological and psychological responses we associate with stress (Endler & Parker, 1990). There is evidence that the state scale of the STAI is sensitive to psychological stress; for example, Rule & Traver (1983) demonstrated a selective increase in the state components of the STAI in response to a stressful social situation, in which male participants were told that their behaviour was going to be filmed while their social skills in front of a female were evaluated (see also Iwamoto et al., 1989; Spielberger et al., 1970). There is also evidence that state STAI scores covary with physiological measures of stress such as pulse rate: Sayette et al. (2001) demonstrated that an instructional stress induction manipulation (the one we employed in Experiment 2) increased both state STAI scores and pulse rate, compared to a no instruction condition.

**Verbatim Instructions**

**Cover Story**

*‘Welcome to this adventure game!*

*You are going to explore a series of caves to collect some treasure; but the treasure is protected by cave spirits, and dangerous things will attack you on the way.*

*We prepared some defences for you to survive.*

*You can wear a helmet to protect yourself from falling rocks...*

*use a torch to drive the bats away...*

*or use a shield to protect yourself from arrows.’*

**Stress Induction**

*‘Before enter the cave:*

*People who have been here before shared fragments of their memories with you.*

*There are some pictures in their memory. If some pictures make you feel very uncomfortable, remember that you can withdraw by closing the window.*

*There is also some sound in their memory. Please turn on the sound.’*

**Instrumental Training**

*‘As you enter the first cave you are going to be attacked, and you must defend yourself by pressing the keys on the main keyboard (z or m). One of these keys repels the bats, whereas the other shields you from the arrows. Because it is dark you do not know which button neutralises which kind of attack. Sometimes the defences will be successful, but often they will not. Now you must try and survive the bats and arrows by using your defences. The sooner you discover the function of the keys the safer you will be.*

*Good luck!*

*The defences need two hands – they will not work if you press both keys at once; so PLEASE DON'T PRESS BOTH KEYS AT ONCE*

**Pavlovian Conditioning**

*‘You have done good work and have survived the first cave - and now your friend has come to help. Your friend has special glasses and can see the attacks coming to you.*

*They will now send you a code of coloured squares to warn you whether an attack is coming, and if so what it is. Your mission in this part of the game is to learn which coloured box indicates whether there is an attack, and what type it is.’*

**PIT test**

*‘Now you will enter the final cave that contains the treasure! Your friend will keep sending you signals to alert you to the type of attack you are facing.*

*You are now asked to press the keys (z or m) to help fend off the attacks, so you can reach the treasure.’*

**EXPERIMENT 2**

**Verbatim Instructions**

**Stress Induction**

*‘After doing the computer task, you will be asked to give a speech about what you like and dislike about your body and physical appearance. You will need to sit in the chair behind you, so we can have the white wall to be the background, look at and talk to this camera (a camera was set about two meters away from the seat). I will turn on this photography light before I leave the room, please do not turn it off. In the speech, you will need to talk about what you like and dislike about your body for at least 3 minutes. Please try to be as open and as honest as possible. A therapist will rate the videotaped speech later. The therapist will rate your openness by analysing how often and in what ways you were defensive. Some common defences include:*

*Denial: Sometimes people may deny something because the truth is hard to accept. Facing reality can be too painful and stressful. For example, when children break things, they often cover their eyes with their hands. Sometimes people refuse to believe that loved ones are passed away.*

*Suppression: When experiencing some pain, people consciously try not to think about it. This reduces the psychological contact with the injury. For example, after a mother lost her child in a car accident, she would quickly change the topic when people mention something about driving. She may also quickly turn the page when she read some news about the car accident in the newspaper.*

*Rationalization: To give an unacceptable emotion, or action some seemingly rational explanation. This is the most common defence people use to cover up their pain or failures in order to maintain peace of mind. For example, if your friend is mean to you, you may rationalise it as them having a bad day rather than letting yourself be hurt by their words.*

*Humour: Sometimes people use humorous language or behaviour to cope with stressful situations, express aggression or desires. For example, jokes about sex, death, and attack are the most popular.*

*These are some examples. In your speech later, we hope you can be as open and honest as possible. Now, if you are ok with it, please sign this consent form to agree to be videoed.’*

**Experiment 1 analysis over six blocks**

***Specific PIT Analysis***

***Difference Scores***

The difference scores for the congruent and incongruent conditions (see Figure S1) indicate congruent scores were higher than incongruent conditions in both groups, and across all six blocks, but that the groups did not differ in their pattern of responding. The impression was supported by the results of a condition (congruent, incongruent) x blocks x group (High, Low) mixed ANOVA, which showed a significant main effect of condition, *F* (1, 70) = 36.352 *p*< .001, *MSE* = 16161188, $\eta_{P}^{2}$ = .143, and of blocks, *F* (5, 350) = 5.447 *p*< .001, *MSE* = 15040 $\eta_{P}^{2}$ = .01. The interaction between condition and blocks was significant, *F* (5, 350) = 2.388, *p*= .038, *MSE* = 24272, $\eta_{P}^{2}$ = .007, but nothing else was significant, largest *F* (1, 70) = 1.442, *p*=.234, *MSE* = 129387, $\eta_{P}^{2}$ = .005 for the main effect of group. Thus there was no sign of any difference in the size of the PIT effect between the two groups.

***Figure S1: Group mean difference scores for congruent and incongruent conditions over the six test blocks of Experiment 1.***

***preCS***

The mean preCS response rates in the congruent and incongruent conditions (see Table S1) did not differ: a conditions (congruent, incongruent) x blocks x groups (stress, positive, neutral) mixed ANOVA showed nothing significant (largest *F* (1,70) = 2.868, *p*= .095, *MSE* = 32527, $\eta_{P}^{2}$ = .003, for the condition x group interaction).

|  | Block 1 | Block 2 | Block 3 | Block 4 | Block 5 | Block 6 |
| --- | --- | --- | --- | --- | --- | --- |
| HIGH Cong | 107.08 (47.97) | 124.58(43.49) | 106.67(39.57) | 82.92 (40.32) | 79.58(28.93) | 95.41(31.02) |
| LOW Cong | 11.25 (4.28) | 55.00 (28.91) | 68.75 (35.05) | 38.33 (25.87) | 101.25 (42.11) | 37.50 (25.48) |
| HIGH Inc | 90.83 (35.95) | 113.33 (37.82) | 65.00 (27.04) | 53.96 (11.99) | 50.83 (12.54) | 55.00 (12.89) |
| LOW Inc | 63.75 (29.47) | 98.33 (38.93) | 44.58 (25.55) | 50.83 (26.83) | 64.17 (33.62) | 72.50 (29.15) |

***Table S1: Group mean preCS response rates for congruent and incongruent in Experiment 1 (rpm; standard error shown in brackets)***

***General PIT Analysis***

***Difference Scores***

The difference scores in the S3 and S4 conditions are presented in Figure S2. Scores for S3 were greater than for S4 condition in both groups and across most of the test blocks. This impression was supported by the results of a conditions (S3, S4) x blocks x groups (High, Low) mixed ANOVA, which showed a significant main effect of condition, *F* (1,69) = 13.538, *p*< .001, *MSE* = 33414, $\eta_{P}^{2}$ = .029, which did not interact with group, or group and block, *Fs* < 1. Nothing else was significant *Fs* < 1. Thus, there was a general PIT effect that did not differ among the groups.

***Figure S2. Group mean difference scores for S3 and S4 over the six test blocks of Experiment 1.***

***preCS***

A condition (S3, S4) x block x group (High, Low) mixed ANOVA was performed on the mean preCS response rates in S3 and S4 (see Table S2), which revealed only a main effect of blocks, *F* (5,350) = 2.38, *p*= .038, *MSE* = 25835, $\eta_{P}^{2}$ = .01, was significant. No other effects or interactions were significant (largest *F* (1,70) = 1.072, *p*= .375, *MSE* = 244929 for the main effect of group.

|  | Block 1 | Block 2 | Block 3 | Block 4 | Block 5 | Block 6 |
| --- | --- | --- | --- | --- | --- | --- |
| HIGH S3 | 27.50 (10.80) | 75.83 (27.05) | 115.41(34.27) | 103.33 (35.71) | 87.50(35.54) | 129.58(41.36) |
| LOW S3 | 7.92 (3.71) | 61.25 (29.13) | 72.08 (32.16) | 68.33 (31.88) | 81.67 (36.55) | 60.83 (33.24) |
| HIGH S4 | 82.50 (27.91) | 114.17 (37.22) | 112.08 (36.73) | 126.67 (43.07) | 70.00 (26.88) | 112.92 (39.26) |
| LOW S4 | 32.50 (14.50) | 66.25 (33.57) | 77.08 (34.63) | 48.33 (26.13) | 92.50 (42.47) | 56.67 (27.00) |

***Table S2: Group mean preCS response rates for S3 and S4 in Experiment 1 (rpm; standard error shown in brackets)***

## **Experiment 2 analysis over six blocks**

***Specific PIT***

***Difference Scores***

The difference scores for the congruent and incongruent conditions are presented in Figure S3. Scores in the congruent condition appeared greater than those in the incongruent condition, and this difference seemed consistent across both groups and blocks. This impression was supported by the results of a condition (congruent, incongruent) x block x group (stress, relax, neutral) ANOVA, which showed a significant main effect of conditions, *F* (1,46) = 35.53, *p*< .001*, MSE* = 25603, $\eta_{P}^{2}$ = .436, which did not interact with group, or group and block, largest *F*  (5,230) = 1.112, *p*=.36*, MSE* = 3031 for the three-way interaction. Nothing else was significant (*Fs* < 1). This suggests that the specific PIT effect did not differ among the three groups.

**Figure S3.** Group mean difference scores for congruent and incongruent conditions over the six test blocks of Experiment 2.

***PreCS***

The group mean preCS response rates in the congruent and incongruent conditions across the six blocks (see Supplementary materials) were subjected to a condition (congruent, incongruent) x block x groups (stress, relax, neutral) mixed ANOVA, which revealed no significant main effects or interactions, largest, *F* (1,46) = 1.251, *p*= .269, *MSE* = 1804for the main effect of conditions.

|  | Block 1 | Block 2 | Block 3 | Block 4 | Block 5 | Block 6 |
| --- | --- | --- | --- | --- | --- | --- |
| STRESS Cong | 46.25 (12.99) | 57.50 (19.19) | 48.75 (15.85) | 51.25 (18.39) | 55.00 (16.27) | 45.63 (17.73) |
| POS Cong | 51.25 (12.99) | 66.88 (21.06) | 60.00 (18.22) | 43.13 (15.96) | 44.38 (16.91) | 37.50 (12.25) |
| STRESS Inc | 69.38 (19.15) | 48.13( 16.70) | 66.25 (19.07) | 46.56 (15.08) | 48.75 (14.43) | 60.63 (20.38) |
| POS Inc | 46.88 (12.46) | 65.00 (17.96) | 51.25 (16.42) | 48.44 (16.13) | 51.88 (16.33) | 51.88 (14.19) |

***Table S3: Group mean preCS response rates for congruent and incongruent in Experiment 2 (rpm; standard error shown in brackets)***

***General Effect***

***Difference Scores***

The difference scores for the S3 and S4 conditions are presented in Figure 9, which suggests that rates during S3 were higher than those during S4, and that this difference was similar in the three groups. This impression was supported by the results of a condition (S3, S4) x block x group (stress, relax, neutral) ANOVA, which showed a significant main effect of condition, *F* (1,46) = 18.263, *p*< .001,  *MSE* = 8381, $\eta_{P}^{2}$ = .284; this did not interact with the group, or group and block, *Fs* < 1, and nothing else was significant (largest *F* (5,230) = 1.162, *p*= .329, *MSE* = 1304 for the interaction between conditions and blocks).

**Figure S4:** Group mean difference scores for S3 and S4 over the six test blocks of Experiment 3.

***PreCS***

The mean preCS response rates in the S3 and S4 conditions (see Supplementary materials) were subjected to a condition (S3, S4) x block x group (stress, relax, neutral) ANOVA which revealed no significant effects or interactions, largest *F* (5,230) = 1.593, *p*= .163, *MSE* = 2342 for the main effect of blocks. This suggests that preCS response rates did not differ between groups or conditions on any of the test blocks.

|  | Block 1 | Block 2 | Block 3 | Block 4 | Block 5 | Block 6 |
| --- | --- | --- | --- | --- | --- | --- |
| STRESS S3 | 72.50 (18.05) | 53.13 (15.56) | 39.38 (12.73) | 43.75 (14.23) | 48.13 (14.10) | 61.88 (16.18) |
| POS S3 | 51.88 (16.33) | 61.25 (16.91) | 55.00 (17.43) | 46.88 (14.51) | 53.13 (17.25) | 44.38 (12.06) |
| STRESS S4 | 57.50 (13.11) | 59.38 (14.66) | 56.25 (15.28) | 42.50 (12.73) | 50.00 (14.35) | 61.25 (15.77) |
| POS S4 | 61.88 (15.59) | 71.25 (18.94) | 40.63 (13.07) | 61.25 (18.26) | 44.38 (15.20) | 51.88 (16.57) |

***zTable S4: Group mean preCS response rates for S3 and S4 in Experiment 2 (rpm; standard error shown in brackets)***

**Experiment 2 analysis over three groups**

**Pavlovian assessment**

The group mean scores for correct and incorrect combinations were 6.52 and 1.50 for Group Stress, 6.36 and 1.51 for Group Positive, and 6.47 and 1.44 for Group Neutral. ANOVA with trial type and group as factors revealed a significant effect of trial type, *F* (1,69) = 581.03, *p* < .001, *MSE* = 1.53, $\eta_{P}^{2}$ = .89, but no effect of group or interaction, *Fs* < 1. The Bayes factor *BF_01_* associated with group was 12.74, strong evidence for the null hypothesis. The mean ratio scores were 0.813, 0.808 and 0.810 for groups Stress, Positive and Neutral respectively; one-way ANOVA confirmed that these scores did not differ, *F* < 1 (*BF_01_* = 8.07). The number of participants in each group with a ratio of less than 0.8 was 5, 7 and 5 in groups Stress, Positive and Neutral respectively; chi-squared confirmed these frequencies did not differ, *p* = .74, and the associated Bayes factor *BF_01_* was 9.90, approaching strong evidence for the null hypothesis. Again there was no evidence that the groups differed in their ability to learn the Pavlovian associations.

***Specific PIT***

The Difference scores for the congruent and incongruent conditions are presented in Figure S5. Scores in the congruent condition appeared greater than those in the incongruent condition, and this difference seemed to be numerically largest in the stress group and smallest in the neutral group, although these differences were very slight. ANOVA with group and condition as factors revealed a main effect of condition, *F* (1,69) = 37.97, *p* < .001, *MSE* = 21.77, $\eta_{P}^{2}$ = .36, but nothing else was significant, *Fs* < 1. *BF_01_* for the effect of group was 10.59, strong evidence in favour of the null model.

The mean preCS scores for congruent and incongruent trials were 50.83 and 61.25 rpm for group Stress, 51.74 and 49.34 rpm for Group Relax, and 56.88 and 62.5 rpm for Group Neutral. These scores did not differ, largest *F*(2,69) = 1.33, *p* =.27, *MSE* = 2.50 for the interaction (BF_01_ = 30.79).

**Figure S5**. Group mean difference scores for congruent and incongruent conditions pooled over the first three test blocks of Experiment 2. Error bars show the standard error of the mean.

***General PIT test***

The difference scores for the S3 and S4 conditions are presented in Figure S6; this suggests that rates during S3 were higher than those during S4; in contrast to the previous experiment, where the positive group showed the numerically smallest general PIT effect, here it seemed to be the largest. Nonetheless, ANOVA with group and condition as factors revealed a main effect of condition, *F* (1,69) = 18.60, *p* < .001, *MSE* = .6.22, $\eta_{P}^{2}$ = .21, but nothing else significant, largest *F* (2,69) = 1.02, *p* = .37, *MSE* = 6.22 for the interaction (*BF_01_* = 7.01). The mean preCS scores for congruent and incongruent trials were 55.00 and 57.71 rpm for Group Stress, 56.04 and 57.91 rpm for Group Relax, and 57.71 and 60.21 rpm for Group Neutral. These scores did not differ, largest *Fs* < 1.

**Figure S6.** Group mean difference scores for S3 and S4 pooled over the first three test blocks of Experiment 2. Error bars show the standard error of the mean.

|  |  | **O1** | **O2** | **O3** | **O4** |
| --- | --- | --- | --- | --- | --- |
| **instrumental** | **condition** | **bats** | **arrows** | **rocks** | **nothing** |
| R1-bats | 1 | S1 | S2 | S3 | S4 |
| R2-arrows | 2 | S2 | S1 | S3 | S4 |
|  | 3 | S1 | S2 | S4 | S3 |
|  | 4 | S2 | S1 | S4 | S3 |
|  | 5 | S3 | S4 | S1 | S2 |
|  | 6 | S3 | S4 | S2 | S1 |
|  | 7 | S4 | S3 | S1 | S2 |
|  | 8 | S4 | S3 | S2 | S1 |
|  |  | **bats** | **rocks** | **arrows** | **nothing** |
| R1-bats | 9 | S1 | S3 | S2 | S4 |
| R2-rocks | 10 | S2 | S3 | S1 | S4 |
|  | 11 | S1 | S4 | S2 | S3 |
|  | 12 | S2 | S4 | S1 | S3 |
|  | 13 | S3 | S1 | S4 | S2 |
|  | 14 | S3 | S2 | S4 | S1 |
|  | 15 | S4 | S1 | S3 | S2 |
|  | 16 | S4 | S2 | S3 | S1 |
|  |  | **rocks** | **arrows** | **bats** | **nothing** |
| R1-rocks | 17 | S3 | S2 | S1 | S4 |
| R2-arrows | 18 | S3 | S1 | S2 | S4 |
|  | 19 | S4 | S2 | S1 | S3 |
|  | 20 | S4 | S1 | S2 | S3 |
|  | 21 | S1 | S4 | S3 | S2 |
|  | 22 | S2 | S4 | S3 | S1 |
|  | 23 | S1 | S3 | S4 | S2 |
|  | 24 | S2 | S3 | S4 | S1 |

**Counterbalancing**

| S1 | red |
| --- | --- |
| S2 | yellow |
| S3 | blue |
| S4 | black |
|  |  |
|  |  |
| R1 | z |
| R2 | m |

**OASIS IMAGES**
